# Expression profiling of chromatin-modifying enzymes and global DNA methylation in CD4+ T cells from patients with chronic HIV infection at different HIV control and progression states

**DOI:** 10.1186/s13148-018-0448-5

**Published:** 2018-02-13

**Authors:** Roberta Nicoleta Bogoi, Alicia de Pablo, Eulalia Valencia, Luz Martín-Carbonero, Victoria Moreno, Helem Haydee Vilchez-Rueda, Victor Asensi, Rosa Rodriguez, Victor Toledano, Berta Rodés

**Affiliations:** 1 Foundation for Biomedical Research of Hospital Universitario La Paz, Madrid, Spain; 2 HIV and Infectious Diseases group, IdiPAZ, Madrid, Spain; 3 Infectious Diseases Department, Hospital Universitario La Paz - Carlos III, Madrid, Spain; 4 Infectious Diseases Department, Internal Medicine, Hospital Universitari Son Espases, IDIPSA, Palma de Mallorca, Spain; 5 Infectious Unit-HIV, Hospital Universitario Central de Asturias (HUCA), Universidad de Oviedo, Oviedo, Spain; 6 Diagnosis and Treatment of Allergic Diseases group, IdiPAZ, Madrid, Spain; 7 Innate Immunity group, IdiPAZ, Madrid, Spain; 8 FIB-Hospital Universitario La Paz- IdiPAZ, Edificio IdiPAZ, Paseo de la Castellana, 261, 28046 Madrid, Spain

**Keywords:** HIV, Progression, Epigenetics, Methyltransferases, HDAC, Chromatin-modifying enzymes, DNA methylation

## Abstract

**Background:**

Integration of human immunodeficiency virus type 1 (HIV-1) into the host genome causes global disruption of the chromatin environment. The abundance level of various chromatin-modifying enzymes produces these alterations and affects both the provirus and cellular gene expression. Here, we investigated potential changes in enzyme expression and global DNA methylation in chronically infected individuals with HIV-1 and compared these changes with non-HIV infected individuals. We also evaluated the effect of viral replication and degree of disease progression over these changes.

**Results:**

Individuals with HIV-1 had a significant surge in the expression of DNA and histone methyltransferases (DNMT3A and DNMT3B, SETDB1, SUV39H1) compared with non-infected individuals, with the exception of PRMT6, which was downregulated. Some histone deacetylases (HDAC2 and HDAC3) were also upregulated in patients with HIV. Among individuals with HIV-1 with various degrees of progression and HIV control, the group of treated patients with undetectable viremia showed greater differences with the other two groups (untreated HIV-1 controllers and non-controllers). These latter two groups exhibited a similar behavior between them. Of interest, the overexpression of genes that associate with viral protein Tat (such as SETDB1 along with DNMT3A and HDAC1, and SIRT-1) was more prevalent in treated patients. We also observed elevated levels of global DNA methylation in individuals with HIV-1 in an inverse correlation with the CD4/CD8 ratio.

**Conclusions:**

The current study shows an increase in chromatin-modifying enzymes and remodelers and in global DNA methylation in patients with chronic HIV-1 infection, modulated by various levels of viral control and progression.

## Background

Chromatin is a highly dynamic structure that, during human immunodeficiency virus (HIV) infection, is altered by the viral integration into the host genome and the host immune response that follows. This virus-host interaction creates an epigenetic environment with a two-way effect. On the one hand, the virus can be modified by chromatin-related events either to increase its replication efficiency or to become transcriptionally inert [1, 2]. On the other hand, T lymphocyte gene expression is modulated to create a cellular environment that favors the establishment of infection, promotes viral replication, and facilitates viral persistence. These chromatin changes are directed by a wide battery of chromatin-modifying enzymes and DNA-associated proteins.

Many phases of the HIV cycle—integration, maintenance of the provirus, and transcriptional activation or silencing—are directly influenced by local chromatin reorganization and abundance of related enzymes. In newly infected cells, the virus is led by chromatin-interacting proteins to targeted DNA sites, especially CpG islands and promoters [3, 4]. Upon integration, the balance between several chromatin-modifying factors creates a more or less permissive environment for viral transcription [5]. The proviral long terminal repeat (LTR) promoter is complexed with two nucleosomes (nuc-0 and nuc-1) that can modify their conformation through binding of multimolecular complexes of histone acetyltransferases (HATs), histone deacetyltransferases (HDACs), and other enzymes. The deposition of nuc-1 is key to the regulation of HIV gene expression. For example, HDAC1, HDAC2, and HDAC3 are main factors that mediate the deacetylation of nuc-1 and compact chromatin [6]. Other proteins that interact with nuc-1 and modulate HIV-1 replication through chromatin reorganization are histone methyltransferases (HMTs) such as SUV39H1 [7], DNA methyltransferases (DNMTs) such as DNMT1 [5], or methyl-CpG binding domain protein 2 (MBD2) [8]. Viral proteins are highly involved in this complex interplay of chromatin rearrangements. Among them, the viral protein Tat is crucial for maintaining the equilibrium of various chromatin-modifying factors for the benefit of viral persistence. Following its synthesis, Tat favors the recruitment of various HATs toward a more permissive chromatin state and active viral replication [9]. Tat itself can be acetylated by some HATs (CBP/p300 and GCN5) to facilitate its interaction with critical cellular proteins and activate viral RNA transcription; without this modification, viral expression would be minimal [10]. At the end of the viral transcription process, Tat is deacetylated by Sirtuin 1 (SIRT1, a class III HDAC) to allow for its recycling [11]. At the same time, however, Tat appears to be a potent inhibitor of SIRT1 in vitro [12]. This second effect of Tat over SIRT1 results in T cell hyperactivation. Although a paradox, this process suggests a complex interaction of Tat with host epigenetic mechanisms, with various effects dependent on Tat concentration and possibly disease stage.

Methyltransferases such as SET domain bifurcated 1 (SETDB1) and protein arginine methyltransferase 6 (PRMT6) also associate with Tat, creating additional levels of viral transcriptional regulation. SETDB1 methylates Tat at lysine 50 and 51 residues, which might initiate transcriptional repression machinery through chromatin remodeling [13], whereas methylation of Tat at lysine 52 and 53 residues by PRMT6 decreases Tat binding to TAR and results in a reduction of HIV transcription [14]. PRMT6 has been suggested to be an HIV restriction factor used by the host. Methyltransferases are also responsible for modifications of DNA methylation. In this regard, the expression of DNA methyltransferases appears to be altered by HIV infection. DNMT1 expression has been reported to increase with HIV-1 infection in vitro and to correlate with gene methylation [15]. Likewise, methyltransferases can also modify proviral DNA. In vitro studies have shown that hypermethylation of the viral LTR promoter silences the virus and helps establish a viral reservoir [8]. However, this effect has been difficult to determine in vivo, because hypomethylated viral genomes appear to predominate in primary blood cells from patients with HIV [16, 17]; thus, further exploration is required to quantify its contribution to latency and reservoirs.

On the other hand, chromatin reorganization drives the plasticity of immune cell populations in response to stimuli. Differentiation of T cell subsets toward helper or regulatory lineages is regulated by DNA methylation events, histones, and other chromatin-modifying enzymes [18]. Key genes such as IFNg, FOXP3, IL17, IL-2, and PD-1, among many others, are modified by several epigenetic mechanisms. In this regard, HIV-1 infection causes important epigenetic changes in the T cell population that result in abnormal expression of proinflammatory cytokines and other immune-related genes. This expression eventually leads to a persistent deregulation of the immune system that is linked to AIDS progression. DNA methylation changes caused by HIV infection have been reported in many of these genes (IFNg, IL-1, PD-1), and these changes are responsible for the broad dysfunction of these cells [19–21].

Despite recognition of the participation of epigenetic mechanisms in HIV-1 infection, there is a paucity of data on the effects of HIV replication levels and the duration of disease on the epigenetic profiles of CD4+ T cells in infected patients. A few in vitro studies mimicking the acute infection in primary PBMCs and CD4+ T cells have observed changes in expression of chromatin-modifying enzymes [22, 23]; however, there are no data on patients with long-term chronic infection.

In this context, the present study aimed to evaluate the expression profiles of chromatin-modifying enzymes in vivo in patients with chronic HIV-1. We also assessed how these factors behaved in various stages of disease by analyzing patients with various phenotypes of viral control and degrees of disease progression.

## Methods

### Study population

A total of 129 individuals were included in the study. Samples from 43 individuals who were HIV-seropositive with various degrees of disease progression and viral control were kindly provided by the HIV BioBank integrated in the Spanish AIDS Research Network [24]; the other 51 individuals who were HIV-seropositive were recruited at Hospital Carlos III in Madrid. Thirty-five HIV-seronegative individuals were also enrolled for the study. Group assignment within the HIV-seropositive individuals was based on their degree of viral control and disease progression. The resulting groups for the final analysis were as follows: 26 chronic progressors treated with cART who presented with undetectable plasmatic viral load for more than a year (hereafter, “cART recipients”); 32 untreated chronic viremic patients with plasma viral loads above 10,000 copies/mL (hereafter, “noncontrollers”); and 36 patients who controlled viremia without treatment (hereafter, “HIV controllers”). Within this latter group, 14 were long-term nonprogressors (LTNPs: untreated patients, with more than 10 years of infection and stable CD4+ T cell count above 500 cells/μL and low levels of viremia) and 22 were elite controllers (EC: untreated patients with undetectable viral load). The patients’ characteristics are described in detail in Table 1.

**Table 1:** Characteristics of the study participants, by group

	HIV-positive			HIV-negative	*p* value
	Noncontroller (without cART) (*n* = 32)	cART recipient (*n* = 26)	HIV-controller (without cART)(*n* = 36)	(*n* = 35)	
Age (years)^a^	35 (25.25–40)	49.5 (40.75–53.75)	44 (39.5–48.5)	49 (41–53)	0.022^b^
Sex (%)					
Men	75	77	68	64	0.399^b^
Women	25	23	32	36	
T cell count, cells/μL^a^					
CD4+	554 (478–713.5)	570 (435–760)	680 (574–1132)	–	0.002
CD8+	865 (736–1148)	694 (433–910)	1031 (622–1470)	–	0.016
Viral load^a^ HIV-1 RNA copies/mL	26,956 (1512.75–54,579.50)	< 20	28.5 (< 20–446.75)	–	< 0.001
Duration after diagnosis (years)^a^	0.16 (0.08–1)	12 (4.75–16)	14 (7.5–19)	–	< 0.001

*cART* combination antiretroviral therapy, *NA* not applicable, *NS* not significant

^a^Data are median (interquartile range, IQR)

^b^Data denote results of comparisons between HIV-positive vs. HIV-negative

All participating individuals provided their informed written consent, and the protocols were approved by the institutional ethics committees. The clinical and epidemiological data provided for patients from the HIV BioBank were included in the adult cohort of the Spanish AIDS Research Network (CoRIS), launched in 2004 [25].

### Sample processing and nucleic acid extraction

Peripheral blood mononuclear cells were obtained from each patient. Subsequently, CD4+ T cells were isolated using the Dynabeads CD4 positive Isolation Kit from Life Technologies, following the manufacturer’s instructions. The purity of the cell fraction was > 98%, as determined by flow cytometry (BD FACSCalibur). Finally, the extraction of RNA and DNA from the CD4+ cells was performed using the AllPrep DNA/RNA/Protein MiniKit (Qiagen). The obtained RNA and DNA were quantified by spectrophotometry using a NanoDrop, purity was also assessed by spectrophotometry, and quality of nucleic acids was analyzed by electrophoresis. The RNA and DNA were conserved at − 80 °C until their further use.

### Quantification of mRNA by real-time PCR

Isolated mRNAs were reverse transcribed with the AMV-RT Access RT-PCR System, (Promega Biotech), using random primers (Biotools B&M Labs, Madrid, Spain) under the following conditions: 95 °C for 5 min before adding the AMV-RT enzyme, followed by incubation at 25 °C for 10 min, 40 °C for 30 min, 48 °C for 30 min, and a final inactivation step of 80 °C for 2 min. Five separate reverse transcription reactions were performed for each patients’ mRNAs, and the resulting cDNAs were pooled for qPCR analysis. The obtained cDNA was amplified by quantitative polymerase chain reaction using TaqMan® technology from Life Technologies. The relative quantification of the genes of interest was performed using TaqMan Array Gene Signature 96-well custom plates, structured as follows: 6 sets of one manufacturing control (18 s), 2 endogenous controls (GAPDH, B2M), and 12 gene expression assays (SETDB1, DNMT3a, DNMT3b, HDAC1, HDAC2, HDAC3, HDAC6, MBD2, HAT1, SUV39H1, PRMT6, SIRT1). One set was occupied in all assays by the same reference sample, which allow us to normalize intra- and inter-assays. The DNMT1 gene expression was analyzed using the TaqMan Gene Expression assay by Life Technologies in an individual format using the same endogenous controls. All the samples were run in duplicate. The assays were performed using an ABI 7500 System, following the manufacturer’s instructions. Relative levels of gene expression were calculated using the ∆∆Ct method as previously described [26].

### Protein extraction and Western blot analysis

Cells pellets were lysed on ice using the following extraction buffer: 50 mM Tris HCl pH = 7,5; 150 nM NaCl; 0,5%SDS; 30 mM PPi; NaF 0,5 M; and 100 μM Na3VO4 20 mM. A protease inhibitors cocktail (Calbiochem) was added before extraction. Cell lysates were separated by 10% SDS-PAGE gel (Mini-PROTEAN TGX gels) and transferred to nitrocellulose membrane (Amersham Protran 0,2 μm, GE Healthcare Life Science).Western blotting was performed using primary antibodies anti DNMT1 (monoclonal antibody DNMT1 clone 60B1220.1, Epigentek, Inc) and DNMT3a (anti-Dnmt3a ab4897, Abcam) and anti ACTB (Actin C-11 sc1615, Santa Cruz Bitotechnology, Inc.) at dilutions 1:1000, 1:1000, and 1:750, respectively. Secondary antibodies conjugated with HRP were used at a dilution of 1:2000, and the reaction was revealed using Amersham ECL Prime Western Blotting Detection Reagent by GE Healthcare Life Sciences, according to the manufacturer’s instructions.

Due to sample limitation, only 27 individuals could be analyzed: 8 HIV-seronegative and 19 HIV-seropositive individuals (3 non-controllers, 10 cART recipients, and 6 HIV-controllers).

### Global DNA methylation analysis

The amount of CpG 5′-methylcytosines in DNA was measured in 100 ng of genomic DNA with the MethylFlash Methylated DNA Quantification kit (Epigentek), following the manufacturer’s instructions. Positive and negative DNA methylation controls were included in the kit. A standard curve was prepared in duplicate with the positive control provided. All DNA samples were measured in duplicate.

### Data analysis

To compare gene expression among the study groups, nonparametric Mann-Whitney *U* and Kruskal-Wallis tests were used when appropriate. For the correlation analyses, we performed the Spearman rank correlation test. Student’s *t* test was used to assess between-group differences in DNA methylation. The association between the categorical variables was evaluated using the Chi-squared test. A *p* value cutoff of 0.05 was used for significance. The statistical analysis was performed using SPSS software 15.0 (SPSS Inc., Chicago, Illinois, USA).

## Results

**Fig. 1:**
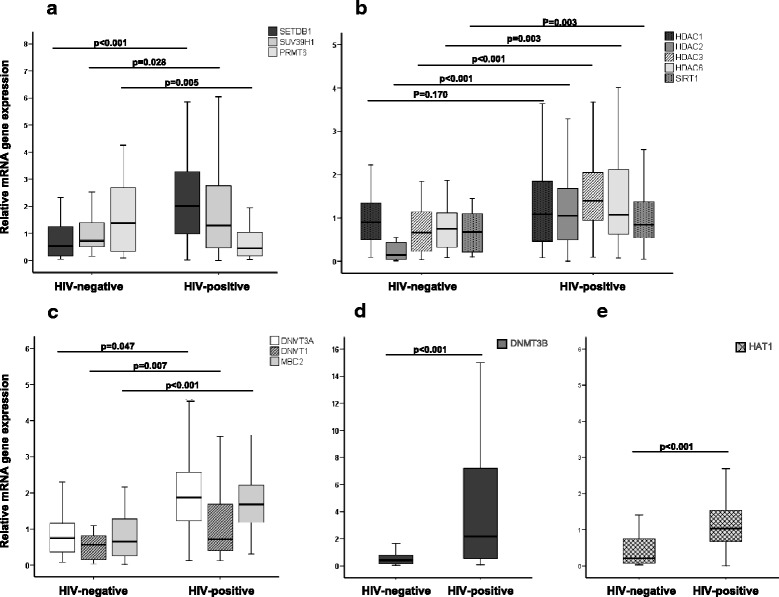
Chromatin-modifying enzyme expression profiling in individuals with chronic immunodeficiency virus type 1 infection-matched HIV-negative controls. **a** Histone methyltransferases (HMTs); (**b**) histone deacetylases (HDACs) Class I, II, and III; (**c**) DNA methyltransferases 1 and 3a and methyl binding protein; (**d**) DNA methyltransferase 3b; and (**e**) histone acetyltransferase 1. Y-exe values represent relative levels of mRNA obtained by the ΔΔCt method. Horizontal bars indicate median values, boxes indicate interquartile range (IQR), and *p* values for each gene are indicated

First, we compared the expression of genes involved in chromatin modification and other epigenetic mechanisms on the CD4+ T cells of patients with HIV versus non-HIV infected individuals. Results showed relevant differences between both groups in almost all the analyzed genes (Fig. 1). In detail, among the analyzed histone methyltransferases (HMTs: SETDB1, SUV39H1, and PRMT6) shown in Fig. 1a, the expression of histone-lysine N-methyltransferase SETDB1 was approximately four times higher in patients with HIV (median = 2.01 vs. 0.52, *p* < .001). The expression of PRMT6, however, was downregulated (three times lower) in individuals who were HIV-positive (median = 0.44 vs. 1.38, *p* = .005). As for the class I, II, and III histone deacetylases (HDAC1, HDAC2, HDAC3, HDAC6, and SIRT1), all showed differences in expression except HDAC1 (Fig. 1b). The highest difference was observed for HDAC2, with over sixfold upregulation in patients with HIV-1 (median = 1.02 vs. 0.15, *p* < .001). The three genes encoding for DNA methyltransferases (DNMT1, DNMT3A, and DNMT3B) were also upregulated in HIV-infected individuals (Fig. 1c, d), with the highest difference observed in DNMT3B (over sixfold higher, median = 2.15 vs. 0.41, *p* < 0.001) (Fig. 1d). The methyl-CpG binding protein MBD2 was also found to be twofold higher in patients with HIV-1 (median = 1.28 vs. 0.64, *p* < 0.001) (Fig. 1c). Finally, histone acetyltransferase 1 (HAT1) also showed upregulation in patients with HIV-1 (Fig. 1e). In a multivariate linear regression analysis, none of these results was significantly confounded by age or gender. Only HIV infection independently associated with changes in gene expression.

**Fig. 2:**
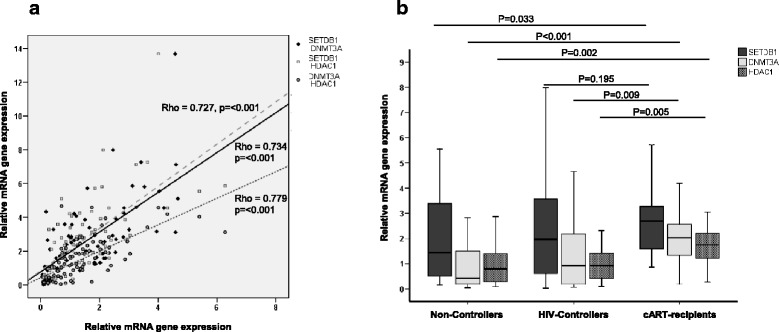
Expression of mRNA levels of SETDB1, DNMT3a, and HDAC1 in patients positive for HIV. **a** Correlation between pairs (SETDB1-DNMT3a, SETDB1-HDAC1, and DNMT3a-HDAC1); (**b**) Differences in SETDB1, DNMT3a, and HDAC1 expression observed in each group of patients positive for HIV. Y-exe values represent relative levels of mRNA obtained by the ΔΔCt method. Horizontal bars indicate median values; *p* values indicate differences for each gene between noncontrollers versus cART recipients and controllers versus cART recipients

We then compared gene expression within the various groups of individuals with HIV-1, classified according to their progression status, treatment, and viral control. The group of combination antiretroviral therapy (cART) recipients showed an overall surge in expression compared with the noncontrollers’ group. We note the elevated expression seen in DNMT3A and DNMTB in cART recipients who control viral replication. All the genes with significant differences between these two groups are shown in detail in Table 2. On the other hand, the HIV controllers behaved highly similar to noncontrollers with no differences in median values for any analyzed gene, except for the expression of DNA methyltransferases, which were slightly increased, although not reaching significance due to wide dispersion of values. Finally, we analyzed associations between genes and observed a positive correlation of expression among SETDB1, HDAC1, and DNMT3A (Fig. 2a). It is in the group of cART recipients that these three genes showed the greatest differences (Fig. 2b). The cART recipients also showed the highest frequency of increased expression in these three genes simultaneously (Table 3).

**Table 2:** Differences in gene expression between cART recipients and noncontrollers

Gene	Median of gene expression (mRNA)		Fold up (cART-recipients vs. noncontrollers)	*p* value
	cART recipients	Noncontrollers		
SETDB1	2.69	1.26	+ 2.15	0.033
PRMT6	0.56	0.23	+ 2.43	0.006
SUV39H1	1.87	0.61	+ 3.07	0.001
DNMT3a	2.01	0.42	+ 4.79	< 0.001
DNMT3b	4.09	0.72	+ 5.68	0.013
MBD2	1.79	0.74	+ 2.42	< 0.001
HDAC1	1.74	0.74	+ 2.35	0.002
HDAC2	1.46	0.52	+ 2.80	0.003
SIRT1	1.08	0.69	+ 1.56	0.001

**Table 3:** Frequency of individuals showing upregulation in all three genes forming the SETDB1-DNMT3a-HDAC1 complex, which interacts with Tat

Type of patient		SEDTB1 + DNMT3A + HDAC1 complex	
		Upregulated	*p* value
HIV negative (*n* = 32)		5 (16%)	< 0.001
HIV positive	Noncontroller (*n* = 26)	8 (31%)	
	cART recipient (*n* = 25)	19 (76%)	
	HIV-controller(*n* = 34)	11(32%)	

Due to missing values, only patients with expression values for SEDTB1, DNMT3A, and HDAC1 were included in this analysis (*n* = 117). *P* value indicates differences between the cART recipients compared to the other subgroups

**Fig. 3:**
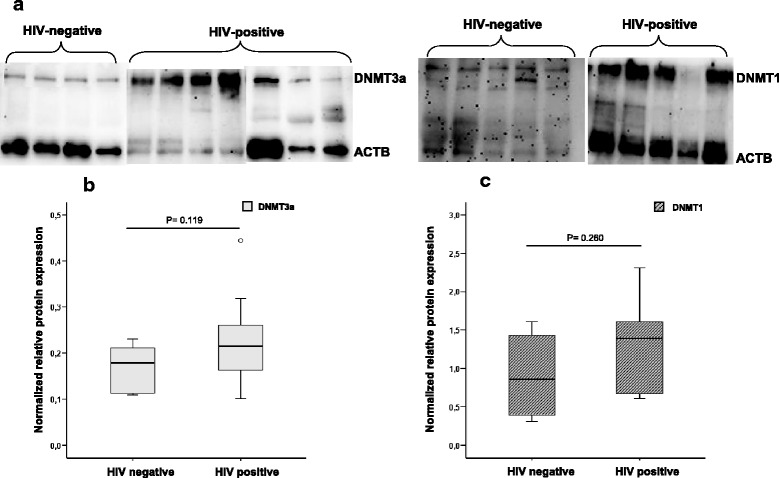
Western blot analysis. **a** Cropped gels/blots obtained by each protein evaluation. **b**, **c** Quantification of the expression of tested proteins and differences between HIV and non-HIV infected individuals: **b** DNMT3a and **c** DNMT1. The optical density of each sample was measured and normalized using an Actin run on the same gel. The data are expressed as relative expression (ratio DNMT/actin). Horizontal bars indicate median values, boxes indicate interquartile range (IQR), and *p* values for each protein are indicated

Western blot analysis showed slightly increased levels of DNMT3a and DNMT1 expression in HIV-positive individuals compared to non-HIV infected, but due to small sample size, differences were not significant. The median values [IQR] for DNMT3a in HIV+ vs. HIV− were as follows: 0.215 [0.162–0.263] vs. 0.178 [0.111–0.214] *p* value = 0.119 (Fig. 3b) and for DNMT1 1.390 [0.640–1.620] vs. 0.860 [0.35–1.52], *p* value = 0.260 (Fig. 3c).

**Fig. 4:**
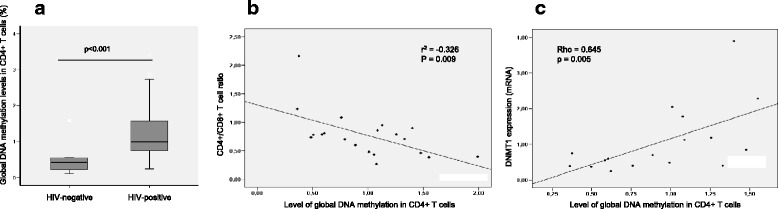
CpG methylation analysis. **a** Percentage of 5′-methylcytosine content in genomic DNA in patients with HIV versus non-HIV infected individuals. Horizontal bars represent mean values and boxes IQR; (**b**) correlation plot between global DNA methylation and CD4+/CD8+ T cell ratio (Pearson correlation test); and (**c**) correlation plot between global DNA methylation and DNMT1 expression in the group of HIV-1 controllers (due to the nature of variables, the nonparametric Spearman correlation test was used)

The analysis of global 5′-methylcytosine in the genomic DNA of CD4+ T cells showed a higher percentage of methylation in the individuals with HIV-1 compared with non-HIV infected individuals (Fig. 4a). Irrespective of HIV status, the global DNA methylation was lower in older individuals (Spearman correlation coefficient rho = − 0.238, *p* = 0.015) and no effect of gender was observed. Within the HIV-1 infected patients, the noncontroller and HIV-controller groups showed a negative correlation between DNA methylation and CD4+/CD8+ T cell ratio (Fig. 4b), whereas this increase in global DNA methylation showed a positive correlation with the expression levels of DNMT1 in the group of HIV controllers (Fig. 4c).

## Discussion

Several studies performed in vitro have analyzed how HIV-1 influences cellular epigenetic mechanisms [22, 23]. Viral infection alters the expression of cellular genes that modify chromatin structure and affect the process of infection. In addition, the viral genome itself is modified by some of these mechanisms, which regulate its level of transcription [8].

Here, we investigated in vivo how the expression of genes that regulate the chromatin environment is modulated in CD4+ T cells depending on the viral control and the degree of progression of patients with HIV.

Overall, the individuals with HIV-1 in our study showed upregulation in all analyzed chromatin-modifying genes compared with the non-HIV infected individuals, with the exception of the PRMT6 gene, which showed downregulation. PRMT6 is able to methylate host proteins such as histone 3, as well as the Tat, Rev, and nucleocapsid viral proteins. This interaction with viral proteins restricts HIV-1 replication [27]. For example, PRMT6 is able to bind HIV-1 Rev, decrease its stability, and attenuate the Rev-Rev response element-dependent export of viral transcripts to cytoplasm, thus negatively affecting HIV replication [28]. Likewise, Tat methylation by PRMT6 decreases viral transcription [14]. Thus, the virus might modulate PRMT6 expression to fine-tune its cycle for its own benefit. An in vitro study mimicking acute HIV-1 infection found PRMT6 to be downregulated immediately after infection, and its expression further decreased over time [22]. Also, knockdown experiments of PRMT6 showed an increase in viral production and faster replication [29]. The lower PRMT6 expression found in our patients with HIV-1 is in accordance with these observations in vitro and might favor viral replication. Otherwise, the expression of PRMT6 has been found to increase in young cells but to decline in replicative and stressed-induced senescence cells [30], suggesting a regulatory role in cell proliferation and senescence. The low PRMT6 expression in patients with HIV might also signify CD4+ T cell exhaustion due to a permanent state of activation, even under antiretroviral treatment. In this regard, the HIV-controllers’ group behaved similarly to noncontrollers, whereas the cART recipients exhibited a slightly higher expression of PRMT6 compared with the other two groups, but still lower than the expression observed in non-HIV infected individuals; this result might indicate residual levels of cell activation despite the success of therapy.

Among the upregulated genes, we can highlight the SETDB1 gene, which not only showed higher expression in individuals with HIV-1, but which also had a positive correlation with DNMT3A and HDAC1 expression. The SETDB1 protein is a histone H3 methyltranferase that, in the context of HIV-1 infection, interacts with and also methylates Tat [13], and its knockdown in vitro produces an increase in viral transactivation. At the same time, SETDB1 has been shown to associate with DNMT3A to promote gene silencing and also with HDAC1, promoting closed conformation of chromatin [31, 32]. It has been proposed that methylation of Tat by SETDB1 facilitates viral silencing by recruiting several gene silencing proteins to remodel chromatin. In our study, we observed that the cART recipients showed higher upregulation of SETDB1, and in most of these patients, this increase in expression was combined with the upregulation of the DNMT3A and HDAC1 genes. This effect was not observed in the other two groups of study patients. Moreover, the group of HIV-controllers behaved similarly to the noncontrollers group. This observation might suggest the recruitment of repression machinery toward the HIV-LTR promoter during antiretroviral treatment, ultimately increasing viral silencing and favoring the establishment of a latent reservoir. It would be interesting to quantify the latent viruses in the cART recipients and compare them with the group of HIV controllers. In addition, the poor performance observed in HIV-controllers regarding SETDB1-DNMT3A-HDAC1, despite viral control, and their closer phenotype to noncontrollers advocates for the beneficial effect of therapy and its extended use for all patients with HIV-1.

SIRT1 is another gene that associates with Tat and that showed differences between the cART recipients and the other two groups of patients with HIV-1. At later phases of infection, Tat inhibition of SIRT1 appears to be critical for inducing cell transformation and apoptosis [33]. The cART recipients, due to the effective therapy, might have lower concentrations of Tat, which in combination with higher expression of SIRT1, could preserve cells and attenuate the effect of Tat in disease progression.

We also analyzed the impact of HIV-1 infection on global DNA methylation. All three groups of patients who were HIV-1 positive had more methylated DNA than the non-HIV infected individuals. An increase in DNA methylation had been observed in vitro within a few hours of HIV-1 infection [23] and in vivo [34]. Likewise, acute HIV infection alters DNA methyltransferase mRNA expression and DNA methyltransferase activity, resulting in an increase in genomic methylation in primary cells [35]; Tat itself has been found to induce overexpression of all three DNMTs [36]. Several studies have linked this increase in methylation to the upregulation and induction in promoter activity of DNMT1 [15, 37]. In our patients, there is an increase in DNMT3A/3B expression, which are the DNA methyltransferases responsible for de novo DNA methylation. However, the observed gains in global DNA methylation show a better correlation with the expression of DNMT1, especially within the HIV-controller’s group, which concurs with the early studies mentioned above and with a previous study published by our group [17], in which we observed an increase in methylation in the LTR region in controllers over time. DNMT1 is usually referred to as the maintenance DNA methyltransferase, but it also exhibits significant de novo activity [38]. In a recent study, Trejbalová and colleagues postulated that transitory activation of T cells contributes to the accumulation of DNA methylation in the viral promoter and that DNMT1 was the enzyme responsible for this [39]. In our HIV-1 controllers, the increase in LTR methylation over time, the correlation of global DNA methylation, and DNMT1 expression would support this hypothesis.

Global DNA methylation also alters cellular function. The changes observed in the methylome of the CD4+ T cells in infected patients also suggest that HIV-1 mediates transcriptional repression affecting cell reorganization. In a recent study, the changes in methylation and increases in methylome variation observed in CD4+ and CD8+ T cells occur in aging T cells and affect the expression levels of genes associated with T cell-mediated immune response, resulting in impaired T cell function [40]. The global DNA methylation observed in our patients with HIV-1 as well as its negative correlation with the CD4+/CD8+ T cell ratio might resemble what is found in an aging immune system.

We would like to acknowledge some limitations in our study. First, the number of analyzed patients in HIV subgroups is small, and it would be of great interest to replicate these findings in more individuals and other HIV populations. Second, global DNA methylation has been measured using an ELISA-based method which serves to identify large differences in DNA methylation. In our study differences between HIV and non-HIV infected individuals were significant; however, other more sensitive methods should be used to detect smaller differences within the HIV subgroups. Finally, due to sample limitation, it was not possible to measure the level of each protein by Western blot to correlate them with mRNA expression in all studied subjects.

## Conclusions

Our study shows alterations in epigenetic mechanisms due to HIV-1 infection in patients with various degrees of progression and viral control. There is an increase in chromatin-modifying enzymes and remodelers, as well as an increase in global DNA methylation. The group of treated patients with controlled viremia differs substantially from the other two groups, which show a similar behavior between them. The fact that patients who have controlled viral replication without treatment show profiles similar to noncontrollers and quite different from cART recipients supports the benefit of therapy.
